# A Puerperal Patient with Leukopenia During Vancomycin Administration: A Case Report and Review of the Literature

**DOI:** 10.3390/ijms26146584

**Published:** 2025-07-09

**Authors:** Lidija Tulic, Katarina Ivanovic, Ivan Tulic, Svetlana Vrzic-Petronijevic, Stefan Ivanovic, Danijela Bratic, Miloš Petronijevic

**Affiliations:** 1 Clinic for Gynecology and Obstetrics, University Clinical Center of Serbia, 11 000 Belgrade, Serbia; tulicivan@yahoo.com (I.T.); vrzic.dr@gmail.com (S.V.-P.); danijelabratic72@gmail.com (D.B.); ordinacija.petronijevic@gmail.com (M.P.); 2 Faculty of Medicine, University of Belgrade, 11 000 Belgrade, Serbia; 3 Obstetrics and Gynecology Clinic “Narodni Front”, 11 000 Belgrade, Serbia; ivanoovic93@gmail.com

**Keywords:** agranulocytosis, leukopenia, vancomycin, antibiotic therapy, puerperium

## Abstract

Antibiotic therapy is essential for managing bacterial infections, but rare yet serious hematological complications such as leukopenia and agranulocytosis may occur. These conditions, although uncommon, require timely diagnosis and intervention, particularly in vulnerable populations such as postpartum patients. This case report describes a 31-year-old puerperal woman who developed agranulocytosis after extended antibiotic treatment for a presumed multidrug-resistant infection. Initially treated with ceftriaxone and metronidazole, her therapy was later escalated to include ciprofloxacin, amoxicillin–clavulanic acid, and vancomycin. *Enterococcus* spp. and *Staphylococcus aureus* were isolated from multiple sites, although no systemic infection was confirmed. Bone marrow findings were consistent with agranulocytosis in the recovery phase. Despite improvements in infection markers, her leukocyte count progressively declined, reaching a nadir of 1.61 × 10^9^/L on the 19th day of therapy. Granulocyte-colony stimulating factor (G-CSF) therapy was initiated, resulting in hematological recovery. The patient was discharged with normal inflammatory markers and leukocyte counts. This case highlights the importance of diagnostic precision, rational antibiotic use, and timely hematologic assessment during prolonged antimicrobial treatment.

## 1. Introduction

Antibiotic therapy represents one of the most impactful advances in modern medicine. However, beyond its life-saving potential, clinicians must be vigilant about the rare but potentially severe adverse events associated with prolonged or broad-spectrum antimicrobial use. Among these, drug-induced leukopenia and agranulocytosis present significant clinical concerns, particularly when they affect vulnerable populations such as postpartum women. These hematologic disorders may emerge due to direct toxicity, immune-mediated mechanisms, or idiosyncratic reactions [1].

The puerperium, being a physiologically dynamic phase characterized by inflammation, tissue remodeling, and hormonal shifts, may further predispose to such events. Importantly, leukocytosis is a normal finding during and after delivery, and thus a decline in leukocyte count can be easily overlooked unless specifically monitored. Clinicians are often faced with the challenge of distinguishing drug-induced cytopenias from infection-related leukocyte changes, especially in the context of suspected or confirmed puerperal infections. As the number of multidrug-resistant pathogens increases, the need for potent antibiotics such as vancomycin has become more prevalent in clinical practice. Understanding the hematologic risks associated with such treatments is crucial to guide monitoring, early recognition, and timely intervention. 

While antibiotics are vital in combating infections, they can cause hematologic side effects such as leukopenia and agranulocytosis—especially concerning in vulnerable populations like postpartum women [2]. Certain antibiotics may suppress bone marrow activity, reducing the production of blood cells (anemia, leukopenia, or thrombocytopenia), while others may cause hemolysis or coagulation abnormalities. Leukopenia is defined as a white blood cell (WBC) count <4 × 10^9^/L, whereas agranulocytosis is a severe reduction in neutrophils (<0.5 × 10^9^/L) [3]. These conditions can result from infections, medications, autoimmune disorders, or bone marrow suppression [4,5]. Leukopenia is rare during pregnancy, as leukocytosis (particularly neutrophilia) is a normal physiological adaptation.

To date, no previously published cases have described vancomycin-induced agranulocytosis in the puerperal period. While several reports have documented antibiotic-associated leukopenia or agranulocytosis in hospitalized adults, these have primarily involved patients with comorbidities such as malignancies, chronic infections, or immunosuppression [6,7,8]. The lack of data on postpartum women highlights the relevance of this case. Physiologic leukocytosis in the puerperium may mask early hematologic deterioration, delaying diagnosis and treatment. This case highlights the need for heightened awareness of hematologic toxicity even in otherwise healthy postpartum patients receiving high-risk antimicrobial agents.

We report a case of a healthy woman whose puerperal period was complicated by severe leukopenia following prolonged antibiotic therapy for a multidrug-resistant bacterial infection.

## 2. Case Presentation

A 31-year-old primigravida woman was admitted at 40 + 2 weeks of gestation for labor because of the spontaneous rupture of fetal membranes. She had no significant personal or family medical history. The pregnancy had been regularly monitored to the term, with no complications. On admission, the patient’s body temperature, heart rate, and blood pressure were within normal physiological range, as well as obstetrics findings and the fetal heart rate. Labor was induced with oxytocin (5 IU Syntocinone in 500 mL 5% Glucose). The patient developed a fever (38.8 °C) six hours after admission. Initial labs revealed leukocytosis (WBC 23.1 × 10^9^/L, neutrophils 90.3%) (Table 1). Empiric therapy with ceftriaxone (2 g/24 h) and metronidazole (500 mg/8 h) was initiated. Twelve hours after admission, the patient delivered a healthy female infant vaginally (3900 g, Apgar scores 9). Prolonged placental separation required manual extraction and lysis.

In the postpartum period, dual antibiotic therapy was continued, and the patient was afebrile. On postpartum day five, she developed a low-grade fever (T 37.2 °C), with CRP 108 mg/L and a WBC drop to 16.8 × 10^9^/L. Microbiological testing identified *Enterococcus* spp. (from uterine cavity, lochia, and episiotomy site) and *S. aureus* (from breast milk), with corresponding antibiograms provided in Supplementary Figures S1–S3. Although these bacteria can be part of normal flora, their presence in lochia, episiotomy, and breast milk, with febrile conditions indicates an active infection. The working diagnosis of “puerperal infection” (endometritis/mastitis) was established in accordance with the clinical picture. Ciprofloxacin (1 g every 12 h) was introduced based on its broad-spectrum activity and the results of the antibiogram, due to suspected urinary tract and wound infection.

Blood cultures (Supplementary Figure S4) and procalcitonin (0.13 ng/mL) levels were analyzed, and both results were negative, indicating no evidence of systemic bacterial infection. Antimicrobial susceptibility testing confirmed multidrug resistance, prompting discontinuation of ceftriaxone and initiation of amoxicillin–clavulanic acid (1.2 g/8 h). On day seven, a temperature spike (38.7 °C), CRP 193.7 mg/L, and WBC 12.3 × 10^9^/L led to consultation with infectious disease specialists. Due to persistent fever despite previous therapy and isolation of *Enterococcus* spp. and *S. aureus* from multiple samples, therapy was escalated to vancomycin (1 g/12 h) per protocol for possible *MRSA* infections. Investigations (chest X-ray, ultrasound, blood cultures, procalcitonin) showed no systemic infection. Bromocriptine was administered for ablactation.

Despite clinical improvement, by the 10th day of initiation of therapy, a significant decline in WBC count (4.52 × 10^9^/L) was noted, accompanied by sustained elevation of CRP (95.3 mg/L). Her WBC dropped progressively, reaching a nadir of 1.61 × 10^9^/L by day 19, with CRP decreasing to 8.43 mg/L. After hematologist consultation, sternal puncture was performed, which revealed agranulocytosis in the recovery phase. The morphology indicated a hypocellular marrow with regeneration of the granulocytic lineage, without signs of dysplasia or infiltration (Supplementary Figure S5). Vancomycin was discontinued and granulocyte-colony stimulating factor (G-CSF) therapy initiated. Leukocyte counts gradually normalized (WBC 3.7 × 10^9^/L), and the patient was discharged.

Coagulation tests and biochemistry remained normal throughout hospitalization. Anticoagulant therapy was initiated because of elevated D-dimer (5.67 mg/L). Vascular surgery consultation was conducted, followed by a color Doppler ultrasound of the lower extremity veins. All follow-up cultures (uterine cavity, lochia, cervix, vagina, episiotomy site, and breast milk) were negative.

**Figure 1:**
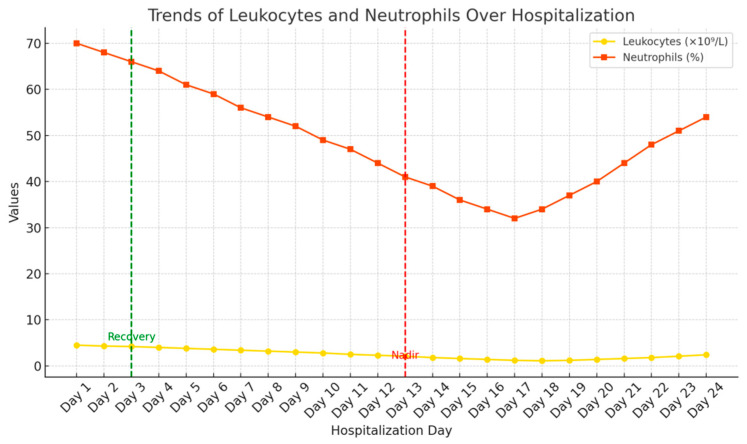
Combined trends of WBC (10^9^/L), Neu (%), Lym (%), and Plt (10^9^/L) throughout the observation period.

**Figure 2:**
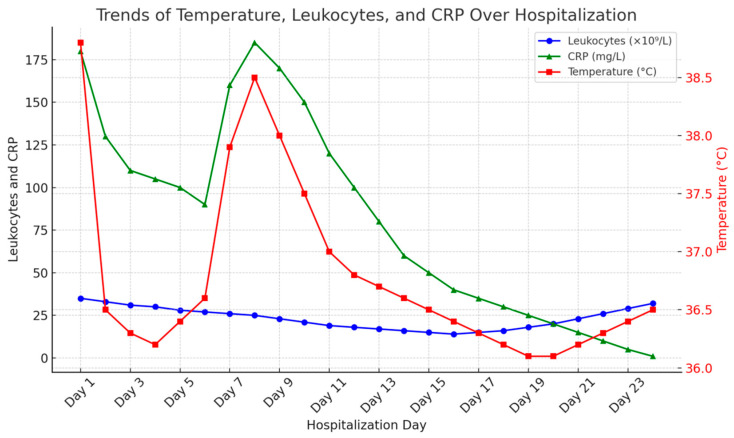
Combined trends of leukocytes, CRP (mg/mL), and temperature (C) throughout the observation period.

**Figure 3:**
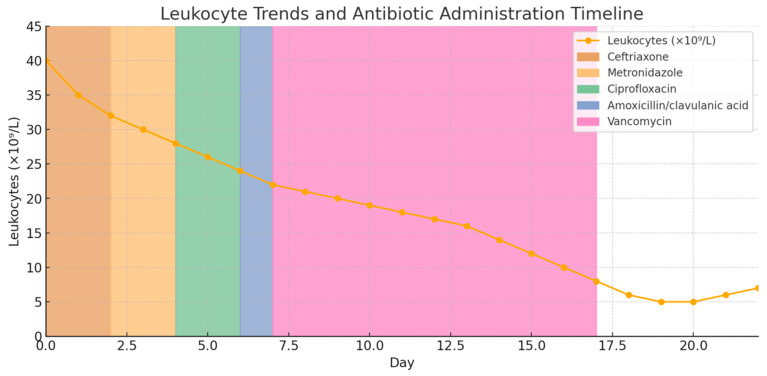
A presentation of antibiotic administration and leukocyte count values during the treatment of the patient.

The following diagrams (Figure 1, Figure 2 and Figure 3) illustrate the temporal relationships between leukocyte count dynamics, inflammatory markers, temperature trends, and antibiotic administration throughout the patient’s clinical course.

## 3. Discussion

### 3.1. Pathophysiological Mechanisms of Antibiotic-Induced Agranulocytosis

Leukopenia and agranulocytosis, indicative of bone marrow suppression, are rare but serious complications associated with the use of certain antibiotics. These conditions are often linked to broad-spectrum antibiotics, especially first-line agents for nosocomial infections and puerperal complications like endometritis or mastitis, particularly in the presence of sepsis [2,5]. A definitive diagnosis of drug-induced agranulocytosis requires a reduction in granulocyte count following the administration of the suspected drug, in the absence of alternative causes of granulocytopenia [7]. In the presented case, Gram-positive bacteria, including *Enterococcus* spp. and *Staphylococcus* spp., were identified from swabs of the uterine cavity, lochia, episiotomy site, and breast milk. Despite these findings, blood cultures were negative and serum procalcitonin levels did not indicate systemic infection. Further diagnostic workup included sternal puncture, which revealed nonspecific agranulocytosis without signs of dysplasia or evidence of systemic infection, despite abnormalities in leukocyte and neutrophil counts. Collectively, these findings point to a drug-induced etiology of agranulocytosis rather than an infection-driven process. The pathophysiology of drug-induced agranulocytosis may involve immune-mediated mechanisms in which drug metabolites act as haptens, binding to neutrophil membranes and triggering antibody formation [8,9]. Additionally, direct suppression of bone marrow granulocytic precursors can occur, particularly with cumulative drug exposure [10]. Clinical presentation can vary from asymptomatic laboratory findings to life-threatening infections, especially when neutrophil counts fall below 0.5 × 10^9^/L. Regular monitoring of hematological parameters is not only important for detecting potential complications early but also for informing decisions about continuing or discontinuing specific antibiotics. In particular, patients undergoing combination or sequential antibiotic regimens with known hematologic risks—such as beta-lactams, vancomycin, and linezolid—should have their complete blood counts monitored bi-weekly. These mechanisms are typically dose-independent and unpredictable, emphasizing the idiosyncratic nature of the adverse reaction [7]. Furthermore, some authors propose that repeated exposure or prolonged therapy increases the likelihood of immune sensitization and subsequent neutrophil destruction. Bone marrow findings often show granulocytic hypoplasia without dysplasia, suggesting a reversible insult rather than permanent marrow injury.

### 3.2. Vancomycin as a Leading Cause of Hematologic Toxicity

Vancomycin, a glycopeptide antibiotic, is widely used for treating Gram-positive infections, particularly those caused by *methicillin-resistant Staphylococcus aureus* (MRSA). Although its nephrotoxicity and ototoxicity are more commonly recognized, hematologic toxicity such as neutropenia is increasingly reported, especially with prolonged therapy.

In this case, the WBC decline occurred after vancomycin administration, a known but infrequent cause of neutropenia. It was observed ten days after the administration of various antibiotic therapies, coinciding with the initiation of vancomycin. The reaction to the drug was an adverse effect and would have occurred regardless of the justification of the application. In the existing literature, vancomycin emerges as a single culprit and one of the two potential culprits responsible for the leukopenia in our patient, while no cases of vancomycin-induced leukopenia/agranulocytosis in puerperal patients have been documented to date [11,12]. In a retrospective cohort study by Lam Philip et al., the incidence of antibiotic-induced neutropenia was evaluated among patients receiving outpatient parenteral antibiotic therapy over a 7-year period. The study reported an overall incidence rate of 2.2 cases per 100 treatment courses. Vancomycin (21/541; 3.9%) and ceftriaxone (10/490; 3.3%) were the most frequently associated intravenous antibiotics, while no cases of neutropenia were observed in patients treated with ceftazidime (45/0; 0%). The incidence of neutropenia related to ampicillin was 2% (98/1), and for penicillin G it was 1.4% (72/1). Notably, all affected patients experienced recovery of neutrophil counts following the discontinuation or completion of antibiotic therapy [13].

In a study of 83 cases of acute antibiotic-induced neutropenia, Holz et al. identified vancomycin as the most frequent cause (18 cases, 21.7%), especially in treatments lasting over two weeks. In addition to vancomycin, commonly implicated antibiotics included ceftaroline, linezolid, penicillin G, and sulfamethoxazole–trimethoprim. Most patients had chronic illnesses and were hospitalized for infectious or hematologic conditions. None were pregnant or postpartum, highlighting the uniqueness of our case. The average duration of antibiotic therapy prior to the onset of neutropenia was 21 days, and recovery of neutrophil counts typically occurred 6 days after discontinuation of the offending agent. The authors emphasized the importance of prompt withdrawal of the suspected antibiotic and the use of G-CSF therapy, which significantly shortened the duration of neutropenia. The timing of the leukocyte decline, the favorable response to G-CSF, and the patient’s prior health status support vancomycin as the likely cause of agranulocytosis [14].

Di Fonzo et al. also reported a case of vancomycin-induced agranulocytosis in a 38-year-old male treated for endocarditis, in whom the absolute neutrophil count dropped to 60/mm^3^ during the second week of therapy. Bone marrow examination revealed a lack of granulocytic precursors without evidence of dysplasia or infiltration. Importantly, the patient tested positive for anti-neutrophil cytoplasmic antibodies (ANCAs) during neutropenia, which reverted to negative following hematologic recovery. These findings support an immune-mediated mechanism of neutrophil destruction rather than direct bone marrow toxicity. Clinical resolution occurred shortly after the discontinuation of vancomycin and administration of G-CSF. This case underscores the importance of considering vancomycin as a potential cause of idiosyncratic agranulocytosis, particularly in the setting of compatible timing and marrow findings [6].

Asai et al. performed a pharmacovigilance analysis of the Japanese Adverse Drug Event Report (JADER) database covering the period from 2004 to 2021. Among over 72,000 reported adverse events, they identified 60 antibiotics, of which 10 were associated with a significant signal for agranulocytosis. Vancomycin was the most frequently implicated, accounting for 72 cases of agranulocytosis among 2428 vancomycin-related adverse event reports, with a reporting odds ratio (ROR) of 3.54 (95% CI 2.73–4.54). The median time-to-onset for vancomycin-induced agranulocytosis was 13 days, emphasizing the importance of hematologic monitoring during mid- to long-term therapy.

These findings quantitatively support vancomycin as the most likely causative agent in this case. This inference is based on the temporal relationship with neutropenia onset, its known hematologic risk profile, and the favorable clinical response following drug discontinuation and initiation of G-CSF therapy [7,14].

### 3.3. Differential Diagnosis and Consideration of Other Agents

Iwazawa et al. documented a similar case in a postpartum woman treated with piperacillin–tazobactam who developed agranulocytosis that reversed rapidly upon cessation of therapy and G-CSF support. These findings highlight a pattern in postpartum patients, suggesting a potential interplay between postpartum immunomodulation and drug hypersensitivity. It remains unclear whether hormonal changes in the puerperium influence susceptibility to such reactions. While no data regarding a potential association with ertapenem are available in the literature, Van Tuyl and colleagues reported a case of meropenem-induced agranulocytosis in a neonate [12,15]. Although rare cases of agranulocytosis induced by amoxicillin–clavulanate acid have been documented, amoxicillin–clavulanate acid is generally regarded as a safe and widely utilized antibiotic [16,17]. Villalba et al. reported a case of agranulocytosis that developed after five days of amoxicillin–clavulanate acid therapy that persisted despite its discontinuation and the introduction of broad-spectrum antibiotics and granulocyte-colony stimulating factor. They ruled out the possibility of an infectious etiology, attributing the agranulocytosis to an immune-mediated response. This conclusion was supported by the widely accepted hypothesis that the drug can activate the immune system via haptens, subsequently inducing direct damage to cells or the bone marrow and leading to agranulocytosis. Despite this case, they concluded that amoxicillin–clavulanate acid remains a generally safe drug for clinical use [18]. In our case report, amoxicillin–clavulanate acid was administered for only two days, with leukocyte levels remaining elevated, preventing any conclusions regarding its impact on the leukocyte lineage.

Agranulocytosis caused by ceftriaxone is rare but can occur in patients receiving high cumulative doses. The resolution of neutropenia occurred within 48 h of ceftriaxone and meropenem being discontinued [19,20]. Hematologic side effects linked to metronidazole are exceedingly rare. Adverse reactions include reversible neutropenia, peripheral neuropathy, and a disulfiram-like reaction when taken with alcohol [21]. In our case, the administration of ceftriaxone for four days and metronidazole for ten days did not result in neutropenia or agranulocytosis. Drug-induced agranulocytosis associated with carbapenem antibiotics is extremely rare [22].

Cimino et al. conducted a comprehensive review of β-lactam-associated neutropenia and reported an incidence of approximately 10% among patients receiving intravenous β-lactams for more than two weeks. They identified both direct myelotoxicity and immune-mediated mechanisms, including cross-reactivity between β-lactams sharing similar R1 side chains. Notably, when patients with neutropenia were switched to a different β-lactam with a dissimilar side chain, only 2 out of 21 cases (9.5%) experienced recurrence, suggesting that careful substitution may be feasible under strict laboratory monitoring [9].

The following table summarizes key reports regarding antibiotic-induced cytopenias, including the class of antibiotics involved, clinical setting, and outcomes (Table 2).

### 3.4. Monitoring, Management, and Role of G-CSF

Management of drug-induced agranulocytosis involves prompt discontinuation of the causative agent, supportive care, and, in severe or prolonged cases, G-CSF therapy. Tesfa et al. emphasized the benefits of G-CSF in hastening marrow recovery and reducing complications [14]. In our case, leukocyte recovery began only after G-CSF administration, supporting its therapeutic role.

Routine CBC monitoring is essential during prolonged antibiotic use, particularly beyond 10–14 days. Guidelines recommend at least weekly monitoring for high-risk agents such as vancomycin, ceftriaxone, and linezolid [23]. Early detection, hematologic consultation, and structured surveillance protocols can prevent complications and improve patient outcomes.

Among the antibiotics administered in this case, vancomycin carries the highest risk of hematologic side effects, with neutropenia and thrombocytopenia reported in approximately 2–8% of cases. The risk is heightened with prolonged intravenous administration, often after prolonged exposure (usually >12 days) [24]. In our case, leukopenia developed nine days after the initiation of antibiotic therapy (WBC 4.52 × 10^9^/L), but the prior use of antibiotics should not be overlooked as a contributing factor. However, vancomycin-induced neutropenia is often reversible, typically resolving within 48–72 h after discontinuation of the drug. In our case, this was not observed, as leukocyte levels continued to decline 48 h post-therapy cessation, with recovery of the leukocyte line occurring only after the introduction of granulocyte-colony stimulating factor [6,7,8,11]. The rationale for G-CSF administration lies in its ability to stimulate bone marrow granulopoiesis and reduce the duration of neutropenia, thereby lowering the risk of opportunistic infections. The cost-effectiveness and safety of this approach have been demonstrated in oncology and infectious disease settings.

## 4. Clinical Takeaway

- In postpartum patients receiving broad-spectrum antibiotics, especially vancomycin, persistent fever and neutropenia should prompt early evaluation for drug-induced agranulocytosis [6,11,15].
- The serial monitoring of leukocyte and neutrophil counts is essential during mid- to long-term antibiotic therapy to detect hematologic complications in a timely manner [7,13].
- Vancomycin-induced agranulocytosis may present with severe neutropenia despite normal inflammatory markers (e.g., CRP) and without signs of bone marrow suppression on peripheral smear [4,10].
- Bone marrow biopsy and immunologic testing (e.g., anti-neutrophil cytoplasmic antibodies) may support the diagnosis of an immune-mediated mechanism [15,18].
- Prompt discontinuation of vancomycin, along with supportive therapy such as G-CSF and broad-spectrum antimicrobial coverage, can lead to full hematologic recovery [9,14].
- Clinicians should be aware of this rare adverse event, particularly in postpartum settings where fever and leukocytosis are often misattributed to infectious causes [2,17].
- Reporting such cases contributes to improved pharmacovigilance and reinforces the need for individualized antibiotic stewardship [11,16,24].

## 5. Conclusions

Vancomycin-induced agranulocytosis, although rare, poses a serious risk in patients undergoing prolonged antibiotic therapy. This case underscores the importance of considering drug-induced cytopenia in the differential diagnosis of postpartum leukopenia, particularly in the context of broad-spectrum antimicrobial use. The puerperium, with its physiologic leukocytosis, may delay recognition of hematologic complications. Regular hematologic monitoring, prompt discontinuation of the offending agent, and early administration of granulocyte colony-stimulating factor (G-CSF) are critical to ensuring rapid recovery and preventing adverse outcomes. Clinicians should remain vigilant for this potentially life-threatening adverse event, especially in otherwise healthy postpartum patients.

## Figures and Tables

**Table 1:** Laboratory data on admission day at maternity ward.

WBC	23.1	10^9^/L
Neu	90.3	%
Lymph	5.3	%
Eosin	0.025	%
Baso	0.138	%
Mono	4.22	%
Rbc	4.27	10^12^/L
Hgb	130.0	g/L
Plt	166.0	10^9^/L

**Table 2:** Comparative presentation of findings of similar case reports.

Author	Study Type	Antibiotic(s) Involved	Population/Setting	Main Findings
di Fonzo H. et al. (2018), [6]	Case Report	Vancomycin	Adult patient	Vancomycin-induced agranulocytosis confirmed via bone marrow exam; recovery post-discontinuation
Lam P.W. et al. (2023), [13]	Retrospective Cohort	Vancomycin, Ceftriaxone	Outpatients	Vancomycin linked to highest incidence of neutropenia among IV antibiotics (3.9%)
Villalba A. et al. (2019), [18]	Case Report	Amoxicillin–Clavulanate	Adult patient	Agranulocytosis resistant to drug withdrawal; G-CSF needed
Couto L. et al. (2021), [19]	Case Report	Ceftriaxone	Adult patient	Neutropenia reversed 48 h after discontinuation
Aung Z.Y. et al. (2024), [20]	Case Report	Ceftriaxone, Meropenem	Hospitalized adult	Agranulocytosis resolved after stopping both agents
Holz J.M. et al. (2020), [14]	Case Series	Vancomycin, Linezolid, Penicillin G, TMP-SMX	Hospitalized adults	Vancomycin was the most frequently implicated antibiotic (21.7% of cases); median onset of agranulocytosis was 21 days after initiation. G-CSF shortened the duration of neutropenia in most cases.
Asai Y. et al. (2022), [7]	Pharmacovigilance Study	Vancomycin	JADER database	Vancomycin had the strongest signal for agranulocytosis among all antibiotics analyzed (72 cases; reporting odds ratio (ROR) 3.54, 95% CI 2.73–4.54).
Cimno C. et al. 2020, [9]	Review	β-Lactam-associated neutropenia	Clinical cases	Approximately 10% of patients on prolonged intravenous β-lactam therapy developed neutropenia.

## Data Availability

Data presented in this study are available from the corresponding author upon request.

## Supplementary Materials

The following supporting information can be downloaded at: https://www.mdpi.com/article/10.3390/ijms26146584/s1.

## Abbreviations

The following abbreviations are used in this manuscript:

WBC	White Blood Cell count
Neu	Neutrophils
Lymph	Lymphocytes
Eosin	Eosinophils
Baso	Basophils
Mono	Monocytes
Rbc	Red Blood Cells
Hgb	Hemoglobin
Plt	Platelets
CRP	C-Reactive Protein
G-CSF	Granulocyte-Colony Stimulating Factor
MRSA	Methicillin-Resistant Staphylococcus aureus
CBC	Complete Blood Count
IU	International Units
