# Supplementary material for: A Puerperal Patient with Leukopenia During Vancomycin Administration: A Case Report and Review of the Literature
Source: Int J Mol Sci. 2025 Jul 9;26(14):6584. doi: 10.3390/ijms26146584 (PMC12295436; doi:10.3390/ijms26146584)
Supplement: Supplementary file 1 [file ijms-26-06584-s001.zip › ijms-3727467-supplementary.pdf]

International Journal of
Molecular Sciences

MDPI

Supplementary Materials

The following supporting figures are provided by the authors.

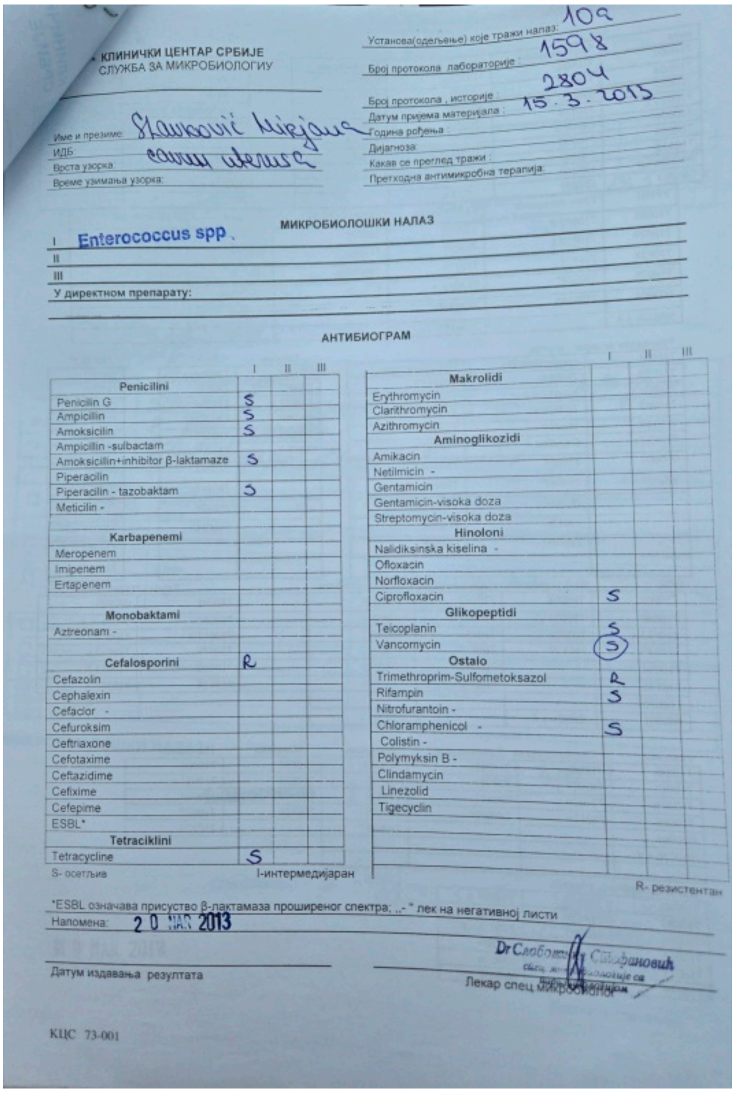

КЛИНИЧКИ ЦЕНТАР СРБИЈЕ
СЛУЖБА ЗА МИКРОБИОЛОГИЈУ

Установа(одељење) које тражи налаз: 109
Број протокола лабораторије: 1598
Број протокола, историје: 2804
Датум пријема материјала: 15. 3. 2013
Година рођења:
Дијагноза:
Какав се преглед тражи:
Претходна антимикробна терапија:

Име и презиме: Stanković Mirjana
ИДБ:
Врста узорка: cavum uterusa
Време узимања узорка:

МИКРОБИОЛОШКИ НАЛАЗ

I Enterococcus spp.
II
III
У директном препарату:

АНТИБИОГРАМ

Penicilini	I	II	III
Penicilin G	S		
Ampicillin	S		
Amoksicilin	S		
Ampicillin -sulbactam			
Amoksicillin+inhibitor β-laktamaze	S		
Piperacilin			
Piperacilin - tazobaktam	S		
Meticilin -			
**Karbapenemi**			
Meropenem			
Imipenem			
Ertapenem			
**Monobaktami**			
Aztreonam -			
**Cefalosporini**	R		
Cefazolin			
Cephalexin			
Cefaclor -			
Cefuroksim			
Ceftriaxone			
Cefotaxime			
Ceftazidime			
Cefixime			
Cefepime			
ESBL*			
**Tetraciklini**			
Tetracycline	S		

Makrolidi	I	II	III
Erythromycin			
Clarithromycin			
Azithromycin			
**Aminoglikozidi**			
Amikacin			
Netilmicin -			
Gentamicin			
Gentamicin-visoka doza			
Streptomycin-visoka doza			
**Hinoloni**			
Nalidiksinska kiselina -			
Ofloxacin			
Norfloxacin			
Ciprofloxacin	S		
**Glikopeptidi**			
Teicoplanin	S		
Vancomycin	S (circled)		
**Ostalo**			
Trimethroprim-Sulfometoksazol	R		
Rifampin	S		
Nitrofurantoin -			
Chloramphenicol -	S		
Colistin -			
Polymyksin B -			
Clindamycin			
Linezolid			
Tigecyclin			

S- осетљив I-интермедијаран R- резистентан

*ESBL означава присуство β-лактамаза проширеног спектра; „-" лек на негативној листи

Напомена: 20 MAR 2013

Датум издавања резултата

Dr Слобо[illegible] Ст[illegible]фановић
Лекар спец микробиолог

КЦС 73-001

Figure S1. Antibiogram of *Enterococcus spp*. isolated from uterine cavity.

 https://doi.org/10.3390/ijms26146584

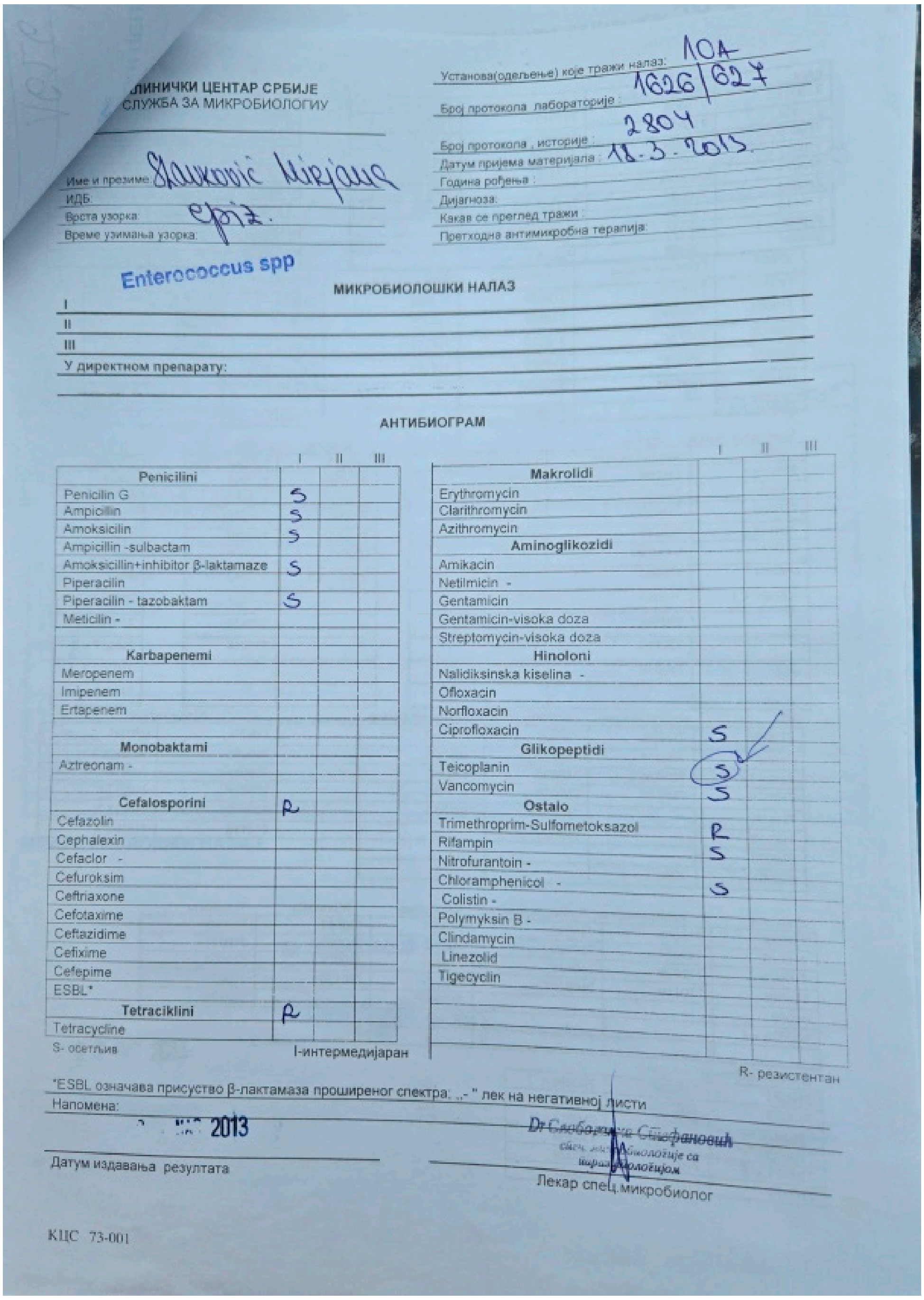

КЛИНИЧКИ ЦЕНТАР СРБИЈЕ
СЛУЖБА ЗА МИКРОБИОЛОГИЈУ

Установа(одељење) које тражи налаз: 10A
Број протокола лабораторије: 1626/627
Број протокола, историје: 2804
Датум пријема материјала: 18-3-2013.
Година рођења:
Дијагноза:
Какав се преглед тражи:
Претходна антимикробна терапија:

Име и презиме: Stanković Mirjana
ИДБ:
Врста узорка: epiz.
Време узимања узорка:

Enterococcus spp

МИКРОБИОЛОШКИ НАЛАЗ

I
II
III
У директном препарату:

АНТИБИОГРАМ

Penicilini	I	II	III
Penicilin G	S		
Ampicillin	S		
Amoksicilin	S		
Ampicillin -sulbactam			
Amoksicillin+inhibitor β-laktamaze	S		
Piperacilin			
Piperacilin - tazobaktam	S		
Meticilin -			
**Karbapenemi**			
Meropenem			
Imipenem			
Ertapenem			
**Monobaktami**			
Aztreonam -			
**Cefalosporini**	R		
Cefazolin			
Cephalexin			
Cefaclor -			
Cefuroksim			
Ceftriaxone			
Cefotaxime			
Ceftazidime			
Cefixime			
Cefepime			
ESBL*			
**Tetraciklini**	R		
Tetracycline			

Makrolidi	I	II	III
Erythromycin			
Clarithromycin			
Azithromycin			
**Aminoglikozidi**			
Amikacin			
Netilmicin -			
Gentamicin			
Gentamicin-visoka doza			
Streptomycin-visoka doza			
**Hinoloni**			
Nalidiksinska kiselina -			
Ofloxacin			
Norfloxacin			
Ciprofloxacin	S		
**Glikopeptidi**			
Teicoplanin	S		
Vancomycin	S		
**Ostalo**			
Trimethroprim-Sulfometoksazol	R		
Rifampin	S		
Nitrofurantoin -			
Chloramphenicol -	S		
Colistin -			
Polymyksin B -			
Clindamycin			
Linezolid			
Tigecyclin			

S- осетљив I-интермедијаран R- резистентан

*ESBL означава присуство β-лактамаза проширеног спектра; „-" лек на негативној листи

Напомена:

[illegible] 2013

Датум издавања резултата

Dr Слободанка Стефановић [illegible]

Лекар спец.микробиолог

КЦС 73-001

Figure S2. Antibiogram of *Enterococcus spp*. isolated from episiotomy.

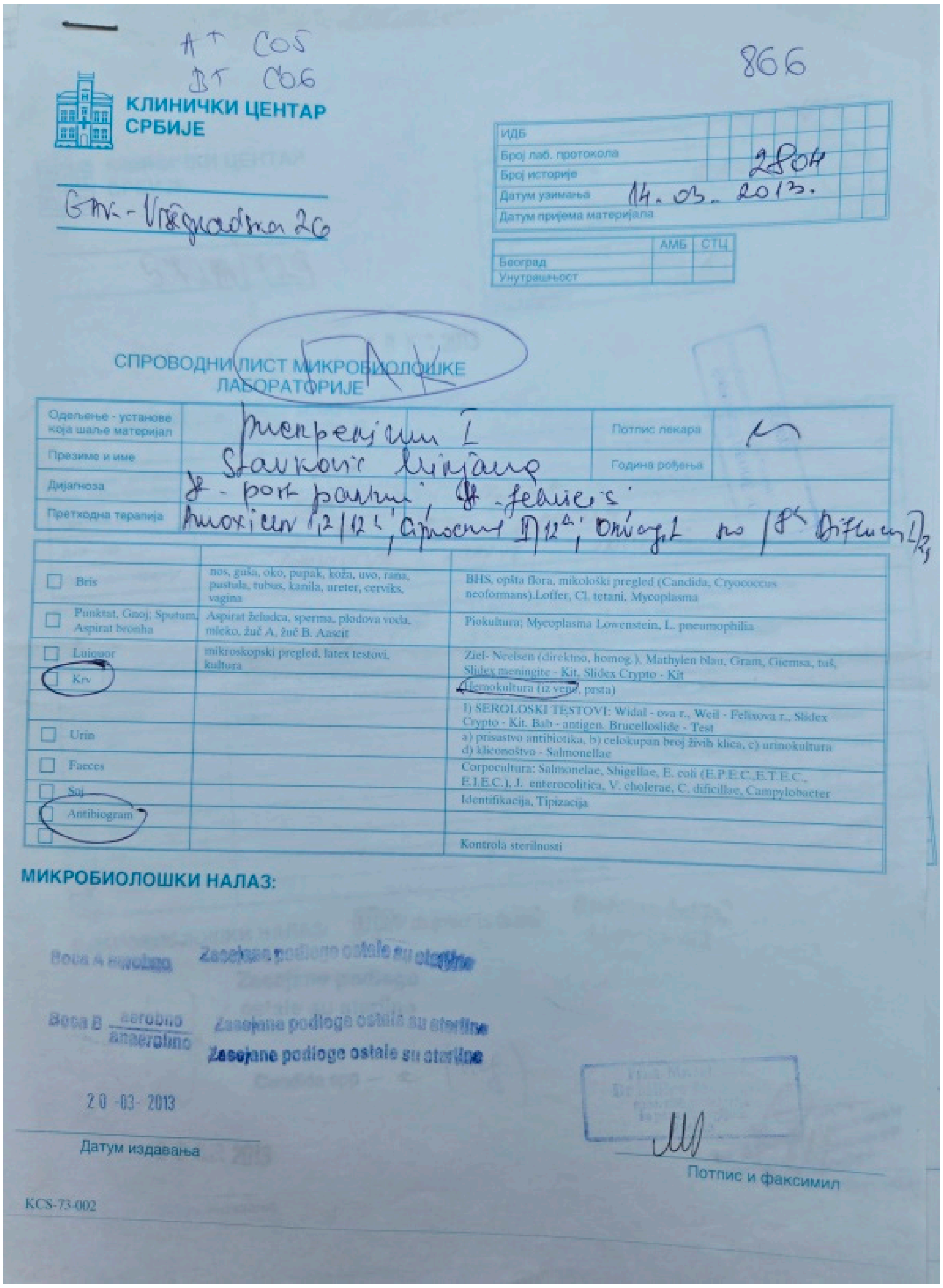
866

КЛИНИЧКИ ЦЕНТАР СРБИЈЕ

ИДБ	
Број лаб. протокола	
Број историје	2804
Датум узимања	14.03.2013.
Датум пријема материјала	

	АМБ	СТЦ
Београд		
Унутрашњост		

СПРОВОДНИ ЛИСТ МИКРОБИОЛОШКЕ ЛАБОРАТОРИЈЕ

Одељење - установе која шаље материјал	[illegible]	Потпис лекара	
Презиме и име	[illegible]	Година рођења	
Дијагноза	[illegible]		
Претходна терапија	[illegible]		

Bris	nos, guša, oko, pupak, koža, uvo, rana, pustula, tubus, kanila, ureter, cerviks, vagina	BHS, opšta flora, mikološki pregled (Candida, Cryococcus neoformans).Loffer, Cl. tetani, Mycoplasma
Punktat, Gnoj; Sputum, Aspirat bronha	Aspirat želudca, sperma, plodova voda, mleko, žuč A, žuč B. Ascit	Piokultura; Mycoplasma Lowenstein, L. pneumophilia
Luiquor	mikroskopski pregled, latex testovi, kultura	Ziel- Neelsen (direktno, homog.), Mathylen blau, Gram, Giemsa, tuš, Slidex meningite - Kit, Slidex Crypto - Kit
Krv		Hemokultura (iz vene, prsta)
		1) SEROLOSKI TESTOVI: Widal - ova r., Weil - Felixova r., Slidex Crypto - Kit, Bab - antigen, Brucelloslide - Test
Urin		a) prisastvo antibiotika, b) celokupan broj živih klica, c) urinokultura d) kliconoštvo - Salmonellae
Faeces		Corpocultura: Salmonelae, Shigellae, E. coli (E.P.E.C.,E.T.E.C., E.I.E.C.), J. enterocolitica, V. cholerae, C. dificillae, Campylobacter
Soj		Identifikacija, Tipizacija
Antibiogram		
		Kontrola sterilnosti

МИКРОБИОЛОШКИ НАЛАЗ:

Boca A aerobno Zasejane podloge ostale su sterilne

Boca B aerobno / anaerobno Zasejane podloge ostale su sterilne

Zasejane podloge ostale su sterilne

20-03-2013

Датум издавања

Потпис и факсимил

KCS-73-002

Figure S3. Antibiogram of blood culture.

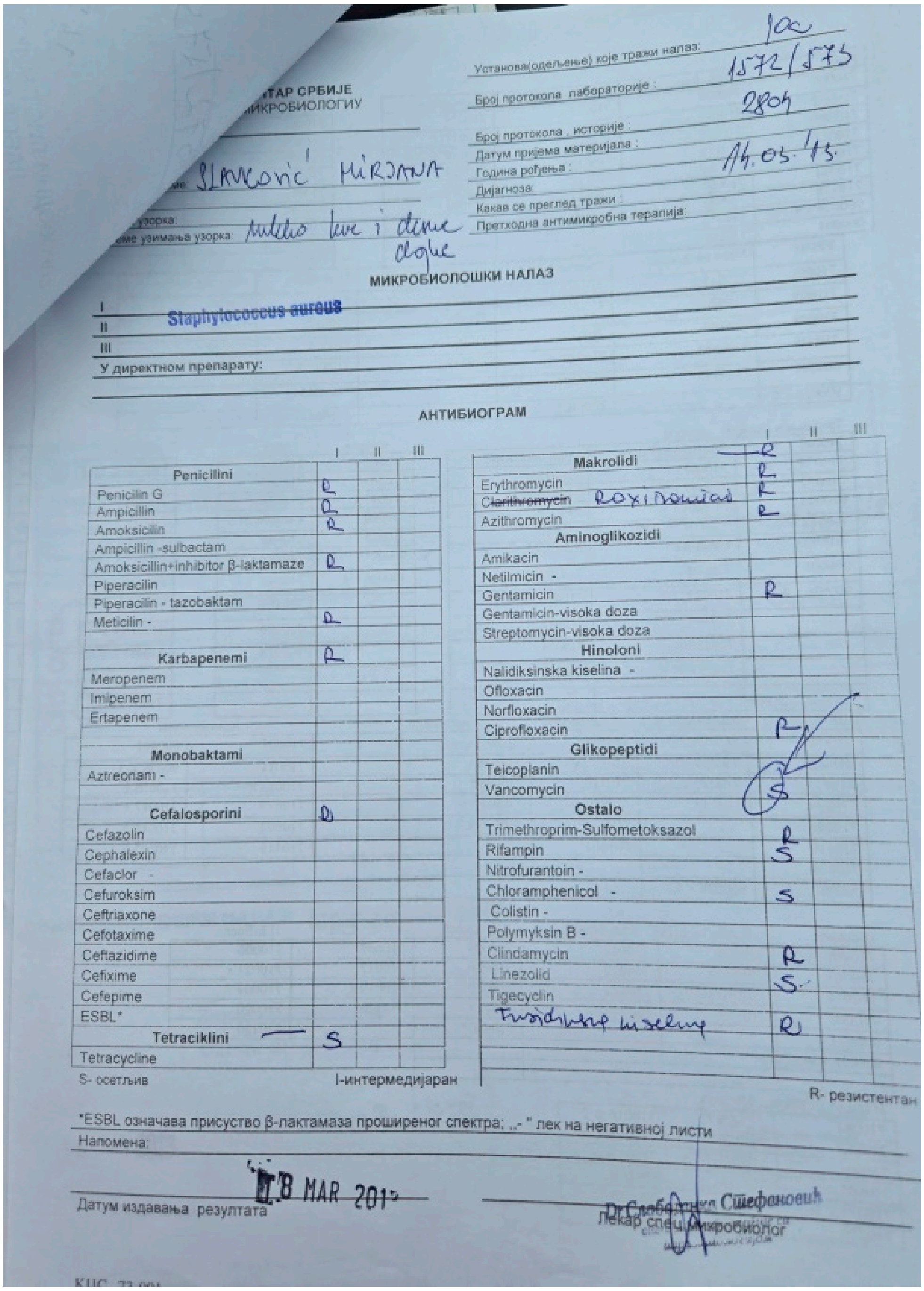

...ТАР СРБИЈЕ
...МИКРОБИОЛОГИЈУ

Установа(одељење) које тражи налаз: [illegible]
Број протокола лабораторије: 1572/573
Број протокола, историје: 2804
Датум пријема материјала: 14.05.'15.
Година рођења:
Дијагноза:
Какав се преглед тражи:
Претходна антимикробна терапија:

...ме: [illegible]
...узорка:
...име узимања узорка: [illegible]

МИКРОБИОЛОШКИ НАЛАЗ

I
II Staphylococcus aureus
III
У директном препарату:

АНТИБИОГРАМ

	I	II	III
**Penicilini**			
Penicilin G	R		
Ampicillin	R		
Amoksicilin	R		
Ampicillin -sulbactam			
Amoksicillin+inhibitor β-laktamaze	R		
Piperacilin			
Piperacilin - tazobaktam			
Meticilin -	R		
**Karbapenemi**	R		
Meropenem			
Imipenem			
Ertapenem			
**Monobaktami**			
Aztreonam -			
**Cefalosporini**	R		
Cefazolin			
Cephalexin			
Cefaclor -			
Cefuroksim			
Ceftriaxone			
Cefotaxime			
Ceftazidime			
Cefixime			
Cefepime			
ESBL*			
**Tetraciklini**	S		
Tetracycline			

	I	II	III
**Makrolidi**	R		
Erythromycin	R		
~~Clarithromycin~~ Roxitromicin	R		
Azithromycin	R		
**Aminoglikozidi**			
Amikacin			
Netilmicin -			
Gentamicin	R		
Gentamicin-visoka doza			
Streptomycin-visoka doza			
**Hinoloni**			
Nalidiksinska kiselina -			
Ofloxacin			
Norfloxacin			
Ciprofloxacin	R		
**Glikopeptidi**			
Teicoplanin			
Vancomycin	S		
**Ostalo**			
Trimethroprim-Sulfometoksazol	R		
Rifampin	S		
Nitrofurantoin -			
Chloramphenicol -	S		
Colistin -			
Polymyksin B -			
Clindamycin	R		
Linezolid	S		
Tigecyclin			
Fusidinska kiselina	R		

S- осетљив I-интермедијаран R- резистентан

*ESBL означава присуство β-лактамаза проширеног спектра; „-" лек на негативној листи

Напомена:

Датум издавања резултата [illegible] MAR 201[illegible]

Др Слободанка Стефановић
Лекар специјалиста микробиолог

Figure S4. Antibiogram of *Staphylococcus aureus* isolated from lochia.

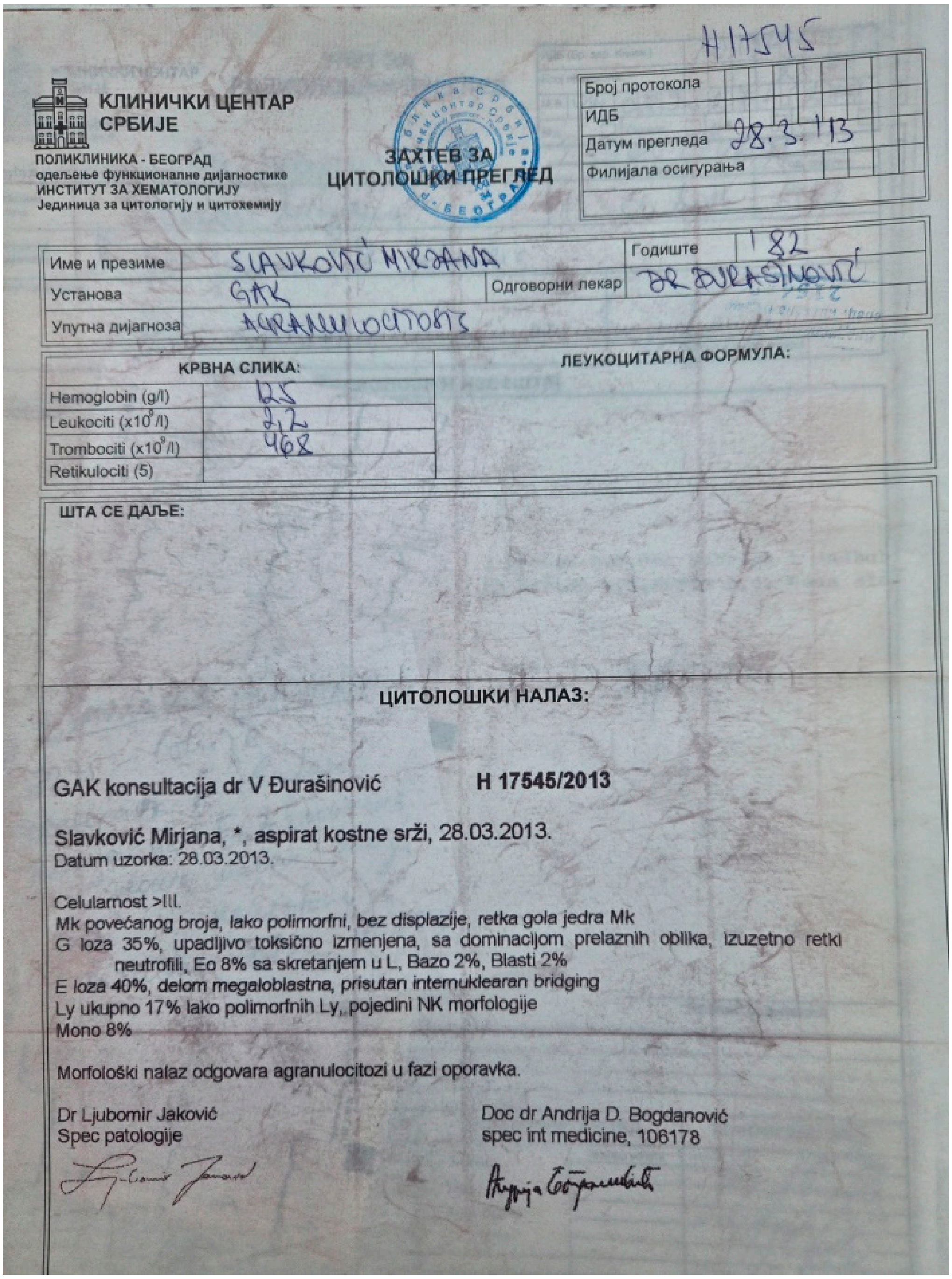

H17545

КЛИНИЧКИ ЦЕНТАР СРБИЈЕ

ПОЛИКЛИНИКА - БЕОГРАД
одељење функционалне дијагностике
ИНСТИТУТ ЗА ХЕМАТОЛОГИЈУ
Јединица за цитологију и цитохемију

ЗАХТЕВ ЗА ЦИТОЛОШКИ ПРЕГЛЕД

Број протокола	
ИДБ	
Датум прегледа	28.3.'13
Филијала осигурања	

Име и презиме	SLAVKOVIĆ MIRJANA	Годиште	'82
Установа	GAK	Одговорни лекар	DR ĐURAŠINOVIĆ
Упутна дијагноза	AGRANULOCITOSIS		

КРВНА СЛИКА:	
Hemoglobin (g/l)	125
Leukociti (x10^9/l)	2,2
Trombociti (x10^9/l)	468
Retikulociti (5)	

ЛЕУКОЦИТАРНА ФОРМУЛА:

ШТА СЕ ДАЉЕ:

ЦИТОЛОШКИ НАЛАЗ:

GAK konsultacija dr V Đurašinović H 17545/2013

Slavković Mirjana, *, aspirat kostne srži, 28.03.2013.
Datum uzorka: 28.03.2013.

Celularnost >III.
Mk povećanog broja, lako polimorfni, bez displazije, retka gola jedra Mk
G loza 35%, upadljivo toksično izmenjena, sa dominacijom prelaznih oblika, izuzetno retki neutrofili, Eo 8% sa skretanjem u L, Bazo 2%, Blasti 2%
E loza 40%, delom megaloblastna, prisutan internuklearan bridging
Ly ukupno 17% lako polimorfnih Ly, pojedini NK morfologije
Mono 8%

Morfološki nalaz odgovara agranulocitozi u fazi oporavka.

Dr Ljubomir Jaković
Spec patologije

Doc dr Andrija D. Bogdanović
spec int medicine, 106178

Figure S5. Cytological finding of bone marrow aspiration.

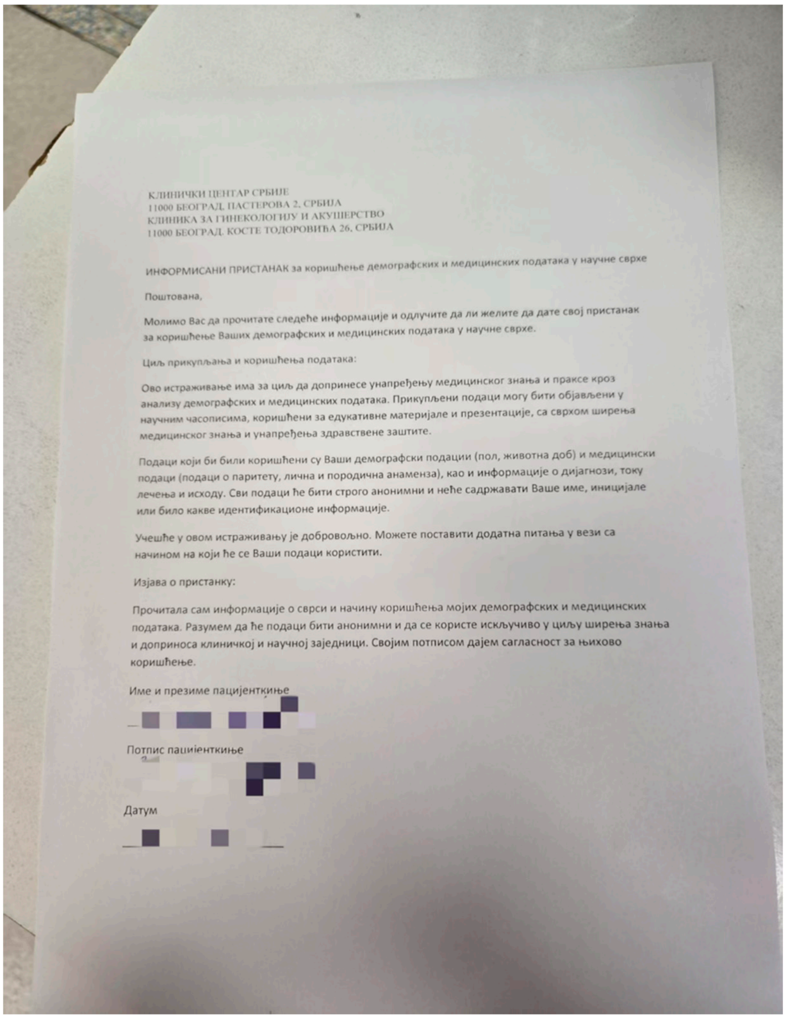

КЛИНИЧКИ ЦЕНТАР СРБИЈЕ
11000 БЕОГРАД, ПАСТЕРОВА 2, СРБИЈА
КЛИНИКА ЗА ГИНЕКОЛОГИЈУ И АКУШЕРСТВО
11000 БЕОГРАД, КОСТЕ ТОДОРОВИЋА 26, СРБИЈА

ИНФОРМИСАНИ ПРИСТАНАК за коришћење демографских и медицинских података у научне сврхе

Поштована,

Молимо Вас да прочитате следеће информације и одлучите да ли желите да дате свој пристанак за коришћење Ваших демографских и медицинских података у научне сврхе.

Циљ прикупљања и коришћења података:

Ово истраживање има за циљ да допринесе унапређењу медицинског знања и праксе кроз анализу демографских и медицинских података. Прикупљени подаци могу бити објављени у научним часописима, коришћени за едукативне материјале и презентације, са сврхом ширења медицинског знања и унапређења здравствене заштите.

Подаци који би били коришћени су Ваши демографски подаци (пол, животна доб) и медицински подаци (подаци о паритету, лична и породична анамнеза), као и информације о дијагнози, току лечења и исходу. Сви подаци ће бити строго анонимни и неће садржавати Ваше име, иницијале или било какве идентификационе информације.

Учешће у овом истраживању је добровољно. Можете поставити додатна питања у вези са начином на који ће се Ваши подаци користити.

Изјава о пристанку:

Прочитала сам информације о сврси и начину коришћења мојих демографских и медицинских података. Разумем да ће подаци бити анонимни и да се користе искључиво у циљу ширења знања и доприноса клиничкој и научној заједници. Својим потписом дајем сагласност за њихово коришћење.

Име и презиме пацијенткиње

[redacted]

Потпис пацијенткиње

[redacted]

Датум

[redacted]

Figure S6. Written informed consent.
